# Epigallocatechin-3-Gallate-Loaded Gold Nanoparticles: Preparation and Evaluation of Anticancer Efficacy in Ehrlich Tumor-Bearing Mice

**DOI:** 10.3390/ph13090254

**Published:** 2020-09-18

**Authors:** Mohamed A. Safwat, Bothaina A. Kandil, Mohamed A. Elblbesy, Ghareb M. Soliman, Nermin E. Eleraky

**Affiliations:** 1 Department of Pharmaceutics, Faculty of Pharmacy, South Valley University, Qena 83523, Egypt; safwat_mohamad@svu.edu.eg; 2 Department of Radiological Science and Medical Imaging, Faculty of Allied Medical Science, Pharos University, Alexandria 21311, Egypt; bothaina.kandil@pua.edu.eg; 3 Department of Medical Biophysics, Medical Research Institute, Alexandria University, Alexandria 21561, Egypt; mohamed.elblbisy@alexu.edu.eg; 4 Department of Medical Laboratory Technology, Faculty of Applied Medical Sciences, University of Tabuk, Tabuk 71491, Saudi Arabia; 5 Department of Pharmaceutics, Faculty of Pharmacy, Assiut University, Assiut 71526, Egypt; nermineleraky@pharm.aun.edu.eg; 6 Department of Pharmaceutics, Faculty of Pharmacy, University of Tabuk, Tabuk 71471, Saudi Arabia

**Keywords:** epigallocatechin-3-gallate, gold nanoparticles, Ehrlich tumor, hemocompatibility

## Abstract

Epigallocatechin-3-gallate (EGCG) is a pleiotropic compound with anticancer, anti-inflammatory, and antioxidant properties. To enhance EGCG anticancer efficacy, it was loaded onto gold nanoparticles (GNPs). EGCG-GNPs were prepared by a simple green synthesis method and were evaluated using different techniques. Hemocompatibility with human blood and in vivo anticancer efficacy in Ehrlich ascites carcinoma-bearing mice were evaluated. EGCG/gold chloride molar ratio had a marked effect on the formation and properties of EGCG-GNPs where well-dispersed spherical nanoparticles were obtained at a molar ratio not more than 0.8:1. The particle size ranged from ~26 to 610 nm. High drug encapsulation efficiency and loading capacity of ~93 and 32%, respectively were obtained. When stored at 4 °C for three months, EGCG-GNPs maintained over 90% of their drug payload and had small changes in their size and zeta potential. They were non-hemolytic and had no deleterious effects on partial thromboplastin time, prothrombin time, and complement protein C3 concentration. EGCG-GNPs had significantly better in vivo anticancer efficacy compared with pristine EGCG as evidenced by smaller tumor volume and weight and higher mice body weight. These results confirm that EGCG-GNPs could serve as an efficient delivery system for EGCG with a good potential to enhance its anticancer efficacy.

## 1. Introduction

Cancer is one of the most debilitating diseases and still ranks as the second-most leading cause of death despite the recent advances in diagnosis and treatment modalities [1]. Approximately one in six deaths is due to cancer and in 2018 it accounted for about 9.6 million deaths [2,3]. An estimated 606,520 persons will die in the United States due to cancer in 2020, which accounts for 1600 deaths per day [4]. It is expected that new cancer cases in 2020 will be about 1,806,590 in the United States [4]. Chemotherapy remains a cornerstone for the management of various cancers despite the serious adverse effects and challenges associated with the use of cytotoxic drugs [5,6]. 

Drugs derived from natural sources (phytochemicals) are more appealing for cancer chemotherapy due to their better safety profile and lower incidence of side effects [7,8]. In this regard, green tea was found to have health benefits against several diseases such as cancer, obesity, diabetes, cardiovascular diseases, and neurodegenerative diseases [9,10]. These beneficial effects are believed to be due to the anticancer, antioxidant and anti-inflammatory properties of the polyphenolic compounds (catechins) present in green tea [11]. (−)-Epigallocatechin-3-gallate (EGCG) is the most abundant biologically active catechin found in green tea [8]. It was found to have antioxidant and anti-inflammatory properties which potentiate its anticancer effects [12,13,14]. EGCG inhibits cell proliferation and induces apoptosis in various types of cancer cells [10,15]. One therapeutically interesting property of EGCG is its selective inhibition of the growth of cancer cells without affecting the surrounding normal cells [16,17]. Despite these intriguing properties, the clinical benefits of EGCG are limited by several shortcomings. For instance, EGCG low stability and degradation in the gastrointestinal tract result in low oral bioavailability [18,19]. It is also susceptible to oxidation and efflux transport from the cells [20]. This, on one hand, shows that high doses of green tea need to be administered to achieve effective concentration of EGCG, which usually results in side effects. On the other hand, it highlights the importance of developing site-specific delivery systems to improve its efficacy and hence, reduce the required dose [21]. 

The literature shows several attempts to overcome these hurdles and enhance EGCG bioavailability and efficacy using several nanoparticle-based formulations such as gold nanoparticles, polymeric nanoparticles, and lipid-based nanoparticles [22,23,24,25,26,27,28]. Among these nanocarriers, gold nanoparticles (GNPs) showed excellent potential to maximize EGCG therapeutic effectiveness while minimizing its side effects. For instance, it was shown that EGCG-capped GNPs were preferentially toxic to tumor cells (Ehrlich’s ascites carcinoma and breast cancer cells) while being safe for normal primary hepatocytes in mice [21]. EGCG-GNPs were also able to increase EGCG efficacy against bladder and prostate cancer in mice and B16F10 murine melanoma cells [7,29,30]. In addition, GNPs offer attractive features for drug delivery applications such as facile synthesis, large surface area, ability to load drugs through physical or chemical bonds with high yield, biocompatibility, and high stability [31,32,33]. 

Several studies have examined the anticancer efficacy of EGCG-GNPs, however, none of them reported the in vivo anticancer efficacy in Ehrlich’s ascites carcinoma. Therefore, the aim of the present study was to prepare and characterize EGCG-loaded GNPs in an attempt to enhance its anticancer efficacy against Ehrlich’s ascites carcinoma. The GNPs were synthesized using a “green” methodology which ensures effective use of EGCG where it serves a triple purpose; the main therapeutic agent, a reducing agent for gold chloride and a stabilizing agent for the formed GNPs. Further, this methodology avoids the use of man-made chemicals as reducing agents, which is environment-friendly, easy to scale up, and limits potentially toxic byproducts produced during GNPs synthesis [32]. EGCG-loaded GNPs were characterized for particle size and morphology, polydispersity index, drug encapsulation efficiency and drug loading capacity. The optimized formulation was characterized for in vitro drug release, storage stability, and compatibility with human blood. Additionally, its in vivo anticancer efficacy was evaluated in mice bearing Ehrlich ascites carcinoma. 

## 2. Results and Discussion

### 2.1. Preparation of EGCG-GNPs

**Figure 1:**
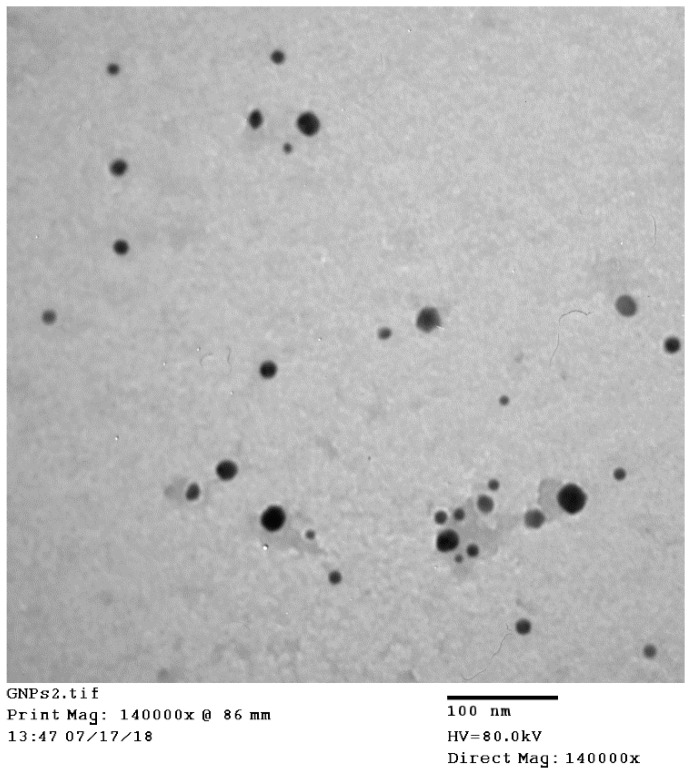
TEM photomicrograph of EGCG-loaded GNPs (formulation F2) showing spherical, discrete particles with an average size of 13.6 ± 5.1 nm.

A green synthesis approach was used for the preparation of EGCG-loaded GNPs where HAuCl_4_ was reduced in aqueous solution using EGCG. This approach is eco-friendly, clean, simple, efficient, and avoids the use of hazardous man-made chemicals [25,32]. EGCG has eight orthophenolic hydroxyl groups, which form complexes with Au^3+^ ions and facilitate their reduction to gold ions to form EGCG-capped GNPs [25]. In addition to acting as a reducing agent, EGCG could also serve as a stabilizing agent for GNPs due to the acidity of EGCG phenolic groups, which results in negative charge at pH 7.4 [25]. The color of the EGCG and HAuCl_4_ mixture changed from pale yellow to purple red within 15 min due to surface plasmon resonance (SPR) phenomenon confirming the formation of gold nanoparticles [34]. Further evidence of the formation of EGCG-GNPs was ascertained from TEM measurements (Figure 1), which shows EGCG-GNPs (formulation F2) as discrete, spherical particles with no aggregation. The size obtained from TEM measurements was 13.6 ± 5.1 nm. This particle size is smaller than that obtained by dynamic light scattering (DLS) measurements (33.8 ± 2.2 nm, Table 1). This is a commonly observed behavior for nanoparticles due to the dry status of nanoparticles used in TEM measurements, which probably results in nanoparticle shrinkage. In contrast, the size obtained by DLS represents hydrated nanoparticles in solution, which explains their bigger size [35,36]. 

### 2.2. Effect of EGCG/HAuCl_4_ Molar Ratio on GNPs Properties

**Figure 2:**
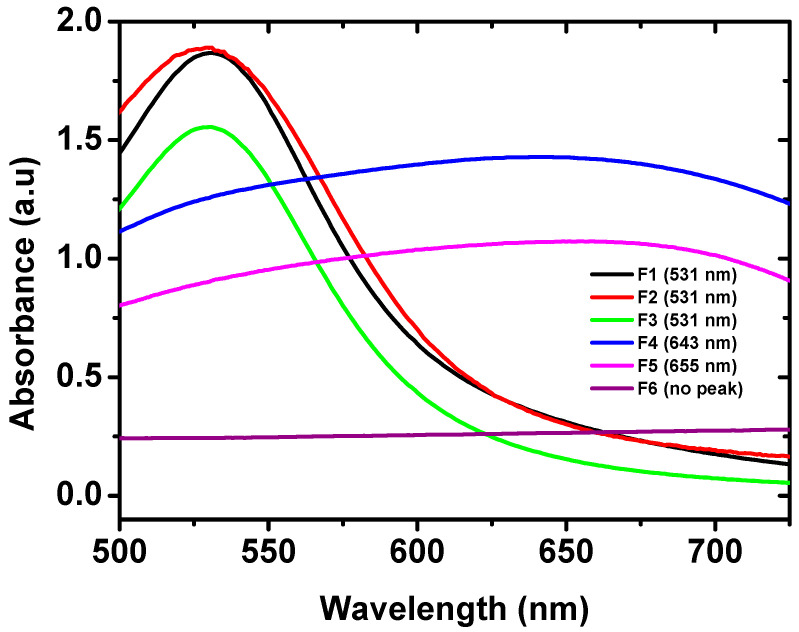
UV-Vis spectra of different EGCG-GNPs formulations in distilled water showing well-defined SPR peaks at λ_max_ of 531 nm for formulations F1, F2 and F3. Formulations F4 and F5 show broad peaks at λ_max_ of 643 and 655 nm, respectively while F6 show no definite peak.

In order to obtain GNPs with optimum properties in terms of drug loading and colloidal stability, the nanoparticles were prepared at various drug/gold chloride molar ratios and their properties were evaluated (Table 1 and Figure 2). As shown in Figure 2, UV-Vis spectroscopy studies represent a well-defined surface plasmon resonance (SPR) peak centered at λ_max_ of 531 nm for F1, F2, and F3. This is in agreement with previous reports [30,37]. These formulations contain, respectively a drug/gold chloride molar ratio of 0.2:1, 0.4:1 and 0.8:1 (Table 1). These SPR absorbance peaks are characteristic of spherical GNPs [25,38]. Increasing the drug/gold chloride molar ratio to 1.6:1 (formulation F4) and 3.1:1 (formulation F5) resulted in decreasing the intensity of the SPR peaks and a red shift to a λ_max_ of 643 and 655 nm, respectively. This red shift might be attributed to GNPs aggregation due to size increase. SPR absorption peaks of GNPs are affected by their size and shape where the increase in size is generally associated with a red shift in the peak [39,40]. A further increase of the ratio to 4.7:1 (F6) was associated with disappearance of the characteristic SPR peak (Figure 2) indicating that GNPs were not formed under these conditions. These results confirm that EGCG concentration as low 109 µM (EGCG/gold chloride molar ratio of 0.2:1) was sufficient for the reduction of gold chloride and formation of gold nanoparticles. This is attributed to the favorable redox potential of EGCG [30]. The GNPs formation was successful for EGCG/HAuCl_4_ molar ratio up to 0.8:1 (F3), after which GNPs started to increase in size and aggregate. The reason for GNPs size increase and aggregation starting from EGCG/HAuCl_4_ ratio of 1.6:1 (F4) is not clear but is probably attributed to the presence of high concentration of EGCG, which leads to saturation of GNPs surface and aggregation. It is therefore recommended that GNPs prepared using EGCG as a reducing and stabilizing agent should be prepared at EGCG/HAuCl_4_ molar ratio not more than 0.8:1. Similar results were observed previously for GNPs prepared using xanthan gum as a reducing agent where increasing the volume of xanthan gum solution resulted in decreasing the intensity of SPR peaks of GNPs [41]. For that system, the maximum intensity of SPR peaks was observed using 5 mL of 1.5 mg/mL xanthan gum solution. 

### 2.3. Effect of EGCG/HAuCl_4_ Molar Ratio on GNPS Size and Polydispersity Index

Nanoparticle size and polydispersity index are known to affect their colloidal stability and in vivo biodistribution and ability to deliver their cargo to specific sites in the body [42,43]. For the successful delivery of anticancer drugs, it was shown that a smaller nanoparticle size facilitates passive accumulation in solid tumors through the enhanced permeability and retention effect [43,44]. The effect of EGCG/HAuCl_4_ molar ratio on nanoparticle size and polydispersity index is shown in Table 1. Except for F5, the increase in the ratio was accompanied by a general increase in the particle size. Thus, increasing the ratio from 0.2:1 (F1) to 0.8:1 (F3) resulted in a change in GNPs size from 26.3 ± 2.4 to 43.8 ± 1.7 nm. However, the change was non-significant (*p* > 0.05). A further increase in the ratio to 1.6:1 (F4) was accompanied by a significant increase in the GNPs size to 332.9 ± 10.5 nm (*p* < 0.05). This highly significant increase in particle size supports the results discussed above for the change in the SPR peaks of GNPs (Figure 2). It is noticed that the particle size of formulation F5 did not follow the trend and was smaller than that of either F4 or F6. Some precipitation was noticed in this formulation, which probably led to the separation of larger particles in the precipitate leaving smaller ones to be measured. Except for F6, the polydispersity index (PDI) of all the formulations was in the range of 0.3–0.5, confirming their reasonable particle size distribution [45].

### 2.4. Effect of EGCG/HAuCl_4_ Molar Ratio on Drug Loading Properties

Results in Table 1 show that EGCG/HAuCl_4_ molar ratio had an important influence on drug encapsulation efficiency and loading capacity. Relatively high encapsulation efficiency and loading capacity of 72 ± 7.7% and 26.4 ± 2.1%, respectively were obtained at molar ratio of 0.2:1 (F1). Both parameters were significantly increased to 92.6 ± 0.6% and 31.7 ± 0.1%, respectively with the increase in EGCG/HAuCl_4_ molar ratio 0.4:1 (F2) (*p* < 0.05). Subsequently, they were decreased upon increasing the ratio to 0.8:1 (F3), yet the change in the loading capacity was non-significant (*p* > 0.05). This indicates that maximum drug loading was achieved for formulation F2 containing 31.65 ± 0.1% EGCG (93 µg/mL EGCG). It is noteworthy that this drug loading capacity is almost four times higher than that reported for EGCG-GNPs prepared previously using 1.0 mM HAuCl_4_ and EGCG/HAuCl_4_ molar ratio around 0.8:1 [25]. This result highlights the importance of the optimization studies presented here to obtain EGCG-GNPs with optimum drug loading capacity. Further increase in the EGCG/HAuCl_4_ molar ratio up to 4.7:1 (F6) resulted in a non-significant change in drug encapsulation efficiency (*p* > 0.05). The decrease in drug encapsulation efficiency with the increase in drug concentration (i.e., increased EGCG/HAuCl_4_ molar ratio) is probably related to reaching maximum drug encapsulation efficiency of around 93% for F2 (EGCG/HAuCl_4_ molar ratio of 0.4:1). After that ratio, the increase in the actually loaded drug amount was not proportional to the increase in the initially used drug concentration leading to deceased drug encapsulation efficiency. This behavior is commonly observed for several other nanocarriers after achieving maximum drug encapsulation efficiency [36,46,47]. It is notable that, although formulations F4–F6 have high drug loading capacity, they have little practical importance due to nanoparticle size increase and aggregation as outlined above in the UV-Vis studies (Figure 2). 

Based on these results formulation F2 was selected for further studies since it has the highest drug encapsulation efficiency (92.6 ± 0.6%), relatively high drug loading capacity (31.7 ± 0.1%) and small particle size (33.8 ± 2.2 nm). Most drug nanocarriers have a limitation of low drug loading capacity, which is generally ≤10% [48]. Therefore, these attributes of formulation F2 are expected to maximize GNPs efficiency as a drug delivery system of EGCG. Furthermore, the potential of a given nanocarrier clinical translation is higher for those having high drug loading capacity since this minimizes the concentration of nanocarrier needed to achieve a clinically relevant drug concentration. A lower nanocarrier concentration not only limits the burden on the body to metabolize and excrete these chemicals, but also improves the colloidal stability of nanocarriers [35,48].

### 2.5. Fourier-Transform (FT-IR) Spectroscopy Studies

**Figure 3:**
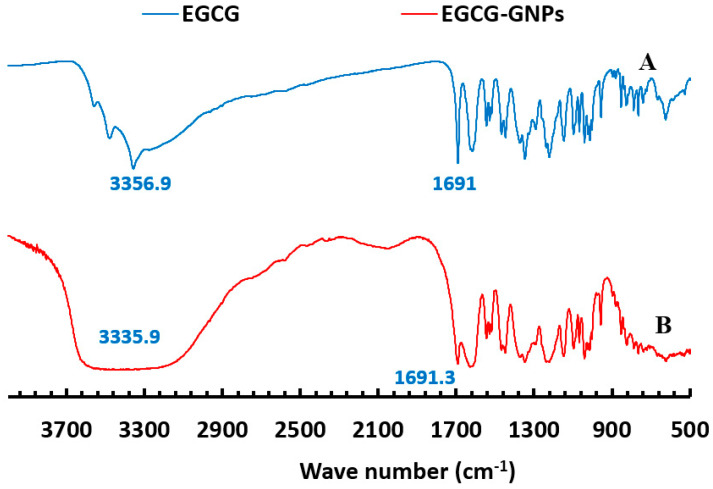
Fourier-transform infrared spectroscopy spectra of EGCG alone (**A**) and EGCG-GNPs (formulation F2) (**B**). The observed absorption peaks confirm the formation of EGCG-loaded GNPs.

FT-IR studies were used to further confirm successful formation of GNPs and loading of EGCG. The FT-IR spectrum of EGCG alone (Figure 3A) shows characteristic absorption bands at 3356.9 cm^−1^ due to −OH and −CH groups stretching, 1691 cm^−1^ due to carbonyl group stretching and a number of bands in the range of 1400–1616 cm^−1^ due to stretching of carbon-carbon bonds in EGCG benzene ring [25,49]. The spectrum of EGCG-GNPs (formulation F2, Figure 3B) shows absorption bands at 3335.9 and 1691.3 cm^−1^, and several bands in the region of 1400–1628.2 cm^−1^, which are ascribed to the stretching vibrations of EGCG. The presence of these bands further confirms the successful loading of EGCG onto the surface of GNPs. 

### 2.6. Differential Scanning Calorimetry (DSC) Studies

**Figure 4:**
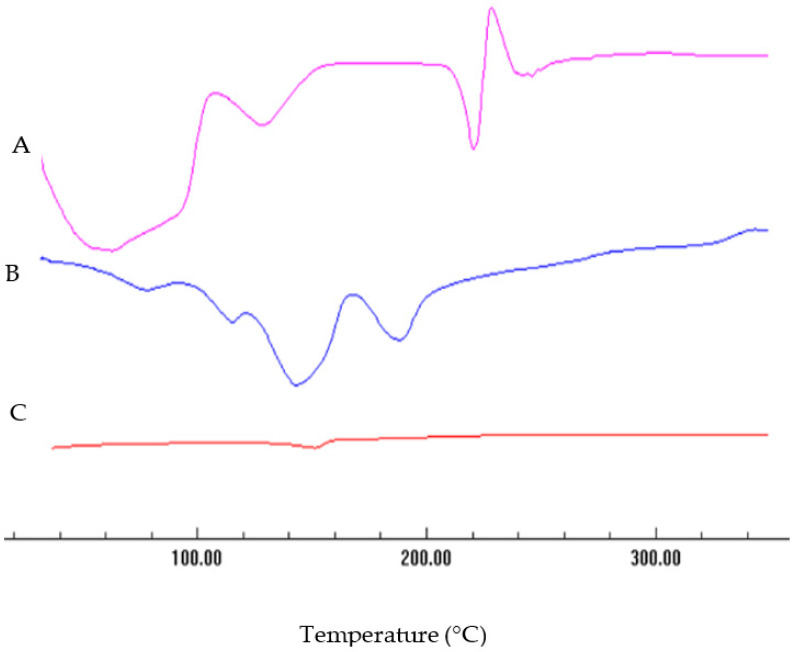
Differential scanning calorimetry thermograms of EGCG alone (**A**), gold chloride alone (**B**) and EGCG-GNPs (formulation F2) (**C**). Disappearance of drug melting endotherm in EGCG-GNPs trace is probably due to drug transformation to an amorphous state.

Figure 4 illustrates the DSC thermogram of EGCG-GNPs (F2) in comparison with the thermograms of EGCG alone and gold chloride alone. The thermogram of EGCG alone (Figure 4A) shows a broad endothermic peak spanning the range of 40–126 °C, probably due to the loss of adsorbed water. This was followed by a sharp endothermic peak at around 220 °C ascribed to the melting of EGCG. This sharp endothermic peak confirms the crystalline nature of EGCG [50]. The thermogram also shows a sharp exothermic peak at 227.7 °C, which is probably attributed to EGCG decomposition. The thermogram of gold chloride alone shows broad endothermic peaks in the range of 100–145 °C, probably due to the loss of adsorbed water and water of crystallization (Figure 4B). Another broad endothermic peak is seen at 188.1 °C and is probably ascribed to melting of gold chloride. The thermogram of EGCG-GNPs reveals disappearance of the characteristic drug peaks (Figure 4C). This is presumably attributed to drug transformation to an amorphous state upon loading onto the surface of GNPs. Other studies showed similar behavior of crystalline drugs loaded onto the surface of GNPs [51,52]. Transformation of the drug to an amorphous state helps in improving its aqueous solubility, which in turn results in better bioavailability and therapeutic efficacy [51].

### 2.7. In Vitro Drug Release Studies

**Figure 5:**
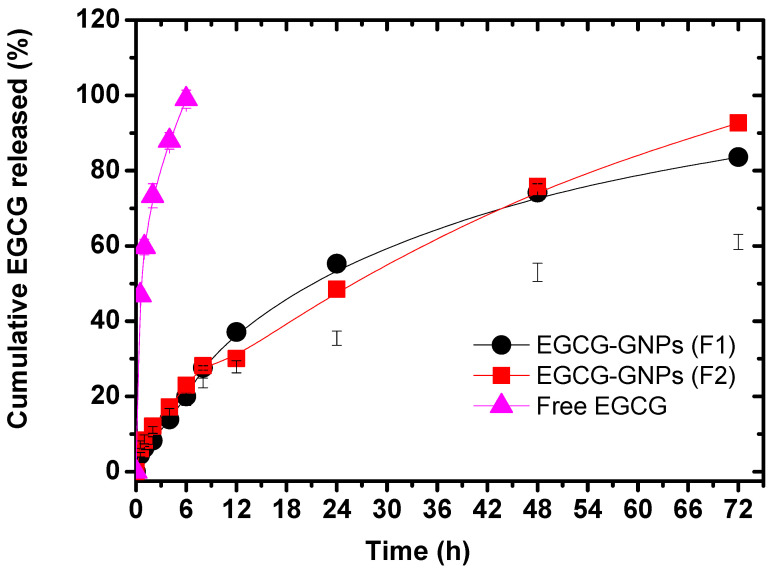
In vitro EGCG release profiles from various GNPs preparations in phosphate buffer pH 7.4 at 37 °C. Loading of EGCG onto GNPs surface significantly slowed down its release rate compared with the drug alone.

EGCG release from its GNPs aqueous solution was studied using the dialysis bag method, a widely used technique to characterize drug release from drug-loaded GNPs [25,36,41]. Aqueous solution of EGCG alone was used as a control to make sure that the drug could easily diffuse through the dialysis membrane. Figure 5 shows that EGCG alone readily and rapidly diffused throughout the dialysis membrane with a cumulative percent released of almost 100% after 6 h. By contrast, the cumulative percent drug released from EGCG-GNPs formulations F1 and F2 were significantly reduced to only 20.17 ± 1.26% and 23 ± 1.9%, respectively (*p* < 0.05), after the same time. The difference between F1 and F2 was non-significant (*p* > 0.05). This pronounced reduction of the drug release rate is presumably attributed to the interactions between EGCG phenolic groups and GNPs surface, thus limiting drug release to the surrounding aqueous medium [25]. Previous studies showed that EGCG-GNPs released much higher drug amounts (i.e., 60%) after the same time and under the same pH and temperature conditions [25] while others showed slower drug release [37]. This difference might be related to the different preparation conditions used in both studies such as concentration of HAuCl_4_ and EGCG/HAuCl_4_ molar ratio. This finding highlights the importance of the preparation conditions of EGCG-GNPs in fine-tuning their drug release properties. The GNPs continued to tardily release their EGCG cargo over an extended period of time where after 72 h, a cumulative percent of 83.6 ± 1.6% and 92.7 ± 2.1% was detected for F1 and F2, respectively, with no significant difference (*p* > 0.05). The ability of nanoparticles to hold their drug cargo for a prolonged period of time is an essential asset to increase their circulation time in the blood and deliver their payload to remote areas, such as in the case of tumors [37]. 

### 2.8. Storage Stability Studies

Aliquots of EGCG-GNPs formulation F2 were stored at 4 °C and at room temperature and at different time intervals up to four months their drug encapsulation efficiency, particle size, polydispersity index, and zeta potential were measured (Table 2). The UV-Vis spectra of the nanoparticles were also recorded and compared with that of freshly prepared ones. Data in Table 2 show that the drug encapsulation efficiency gradually decreased with time for all the samples regardless of the storage temperature. Statistical analysis of the data shows that the decrease in drug encapsulation efficiency for samples stored at 4 °C was non-significant compared with the freshly prepared one for up three months (*p* > 0.05), after which the decrease was significant (*p* < 0.05). In contrast, the samples stored at room temperature showed a significant decrease in the drug encapsulation efficiency (*p* < 0.05), which started after one month and continued for the whole period of study. This decrease in drug content is probably attributed to drug release from the GNPs surface to the surrounding aqueous medium due to the concentration gradient between the nanoparticle surface and the surrounding aqueous medium. It is therefore recommended to store these nanoparticles at temperature not exceeding 4 °C and for not more than three months. Nonetheless, the maximum amount of drug lost from the GNPs after four months of storage at room temperature was small (ca. 10%, Table 2), confirming the acceptable stability of these nanoparticles even at room temperature. 

Change in the size of EGCG-GNPs over time followed the opposite trend. Thus, the change in size for the samples stored at room temperature was non-significant (*p* > 0.05) for the whole period of study. In contrast, all the differences in size for the samples stored at 4 °C were significant (*p* < 0.05) except that between the samples of zero time and four months. However, maximum change in size was about 11 nm (Table 2). While this change is numerically significant, it has little practical significance since the size of the nanoparticles is still small and below 45 nm. The PDI showed a small non-significant change (*p* > 0.05) for all the tested samples. Further, after four months there was no change in the SPR peak for all the samples, which remained at λ_max_ of 528 nm. This confirms that the samples maintained their homogeneity with no aggregation or dissociation during the four-month storage period. 

Zeta potential is one of the most important attributes of nanoparticles since it affects their colloidal stability in solution, as well as their behavior in vivo. Freshly prepared EGCG-GNPs formulation F2 had a zeta potential of −24.9 ± 0.3 mV, indicating their excellent colloidal stability through electrostatic repulsion between the nanoparticles (Table 2) [31]. During the four-month study period the zeta potential ranged from −24.9 ± 0.3 to −28.0 ± 0.6 mV confirming the colloidal stability of the nanoparticles. Some of the changes in zeta potential were statistically significant compared to freshly prepared nanoparticles but they were not practically significant since they were less than −4 mV. 

### 2.9. Hemocompatibility Studies

#### 2.9.1. Hemolysis Studies

**Figure 6:**
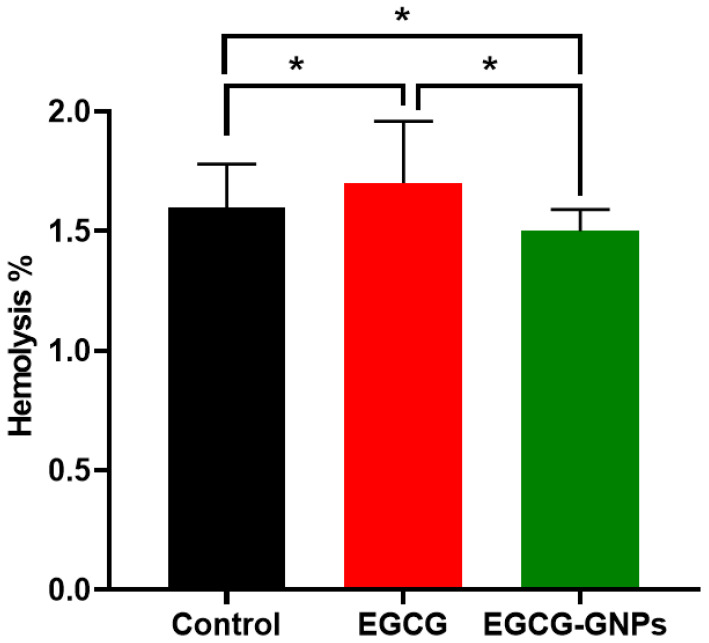
Hemolytic effect of epigallocatechin-3-gallate (EGCG) and epigallocatechin-3-gallate gold nanoparticles (EGCG-GNPs). * Statistically significant difference (*p* < 0.05). Blood hemolysis caused by EGCG or EGCG-GNPs was less than 2% confirming their hemocompatibility.

The hemocompatibility of EGCG-GNPs was tested since it is suggested to be administered by intravenous injection. Following this administration, blood is the first body component that drug-loaded nanoparticles encounter, which highlights the importance of hemocompatibility studies [53]. Many in vitro tests are approved for evaluating the hemocompatibility of NPs, such as hemolysis, and blood coagulation tests [54,55]. Hemolysis is considered to be one of the simple and reliable measurements used for estimating the blood compatibility of NPs [56]. Figure 6 shows that hemolysis percentage due to the incubation of blood with EGCG or EGCG-GNPs was below 2%, confirming their hemocompatibility. The hemolysis caused by pure EGCG (1.7 ± 0.26%) was significantly higher than that observed in the control (*p* < 0.05). The percent hemolysis caused by EGCG is higher than that reported previously, probably due to the different experimental procedures [7]. Interestingly, the hemolysis caused by EGCG-GNPs solution (1.5 ± 0.09%) was significantly smaller than that observed in the control (1.6 ± 0.18%) and EGCG samples (*p* < 0.05). The lower hemolysis observed for EGCG-GNPs might be due to their negative surface charge (Table 2). Previous studies showed that blood hemolysis caused by GNPs was concentration- and surface charge-dependent, where neutral and negatively charged nanoparticles had lower hemolysis compared to positively charged ones [57]. 

#### 2.9.2. Effect of EGCG and EGCG-GNPs on Prothrombin Time and Partial Thromboplastin Time

**Figure 7:**
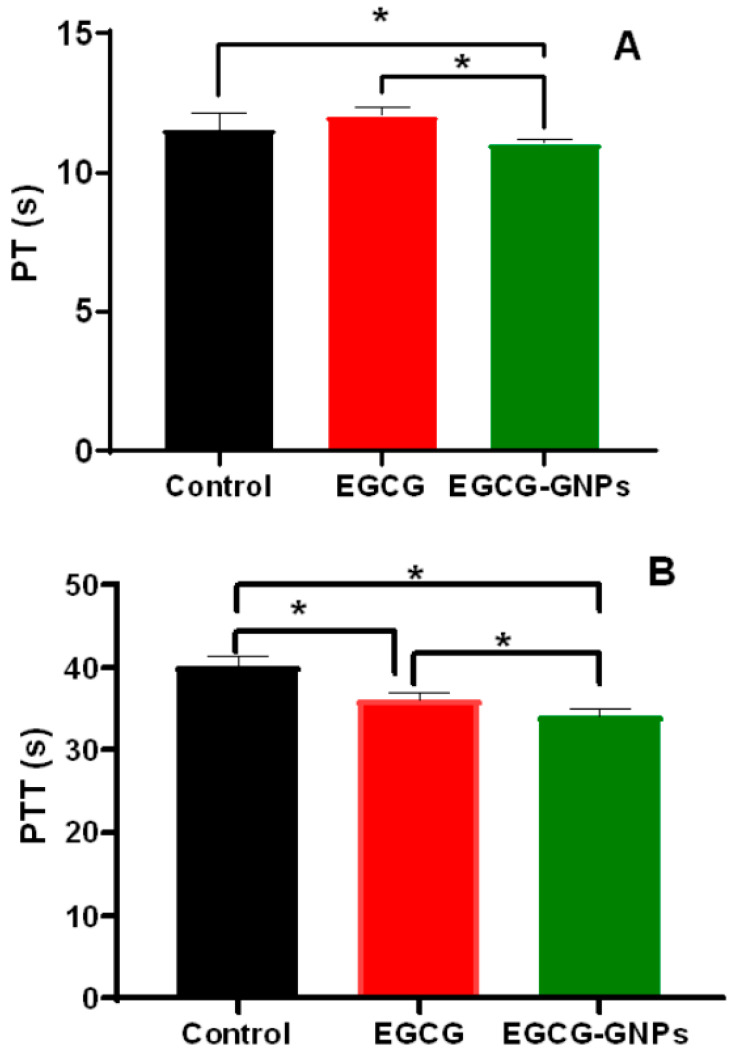
Effect of epigallocatechin-3-gallate (EGCG) and epigallocatechin-3-gallate- gold nanoparticles (EGCG-GNPs) on clotting of human blood. (**A**) Prothrombin time (PT) of human blood after incubation with EGCG and EGCG-GNPs. (**B**) Partial thromboplastin time (PTT) of human blood after incubation with EGCG and EGCG-GNPs. * Statistically significant difference (*p* < 0.05). PT and PTT for blood samples incubated with either EGCG or EGCG-GNPs were within the normal range (PT ≤ 13.5 s and PTT ≤ 40 s), which further confirms their hemocompatibility.

Next, we evaluated the effect of EGCG and EGCG-GNPs on prothrombin time (PT) and partial thromboplastin time (PTT), which are frequently used to assess blood clot formation. GNPs could affect the coagulation system by interacting with either plasma coagulation factors or blood cells [58,59,60]. PT for the blood incubated with EGCG was not significantly different from that of the control (*p* > 0.05). In contrast, PT of the blood incubated with EGCG-GNPs was significantly lower than that of the control or EGCG (*p* < 0.05) (Figure 7A). Figure 7B shows that PTT of the blood incubated with EGCG-GNPs or EGCG was significantly lower than that of the control (*p* < 0.05). However, it could be concluded that neither EGCG nor EGCG-GNPs had abnormal effects on PTT and PT since their values lie within the normal range (PT ≤ 13.5 s and PTT ≤ 40 s) [61]. This confirms that the prepared EGCG-GNPs are biocompatible and free of deleterious effects on the blood clotting system.

#### 2.9.3. Effect of EGCG and EGCG-GNPs on Complement Protein (C3)

**Figure 8:**
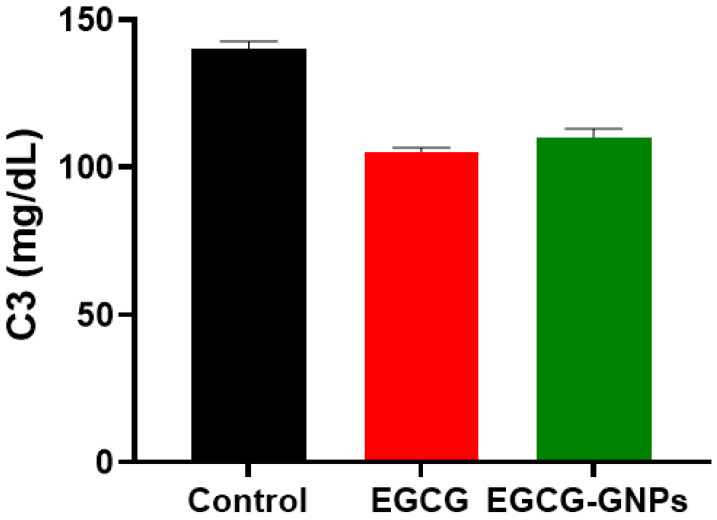
Assessment of the effect of epigallocatechin-3-gallate (EGCG) and epigallocatechin-3-gallate gold nanoparticles (EGCG-GNPs) on the complement component 3 concentration, in vitro. The non-significant decrease in C3 concentration caused by EGCG or EGCG-GNPs confirms the biocompatibility of the prepared EGCG-GNPs.

Complement component 3 (C3) is a protein that has an essential role in the complement system and innate immunity [62]. Interaction of intravenously injected nanoparticles with plasma proteins could modulate their pharmacokinetics and biological performance [63,64]. Figure 8 shows the in vitro effect of EGCG and EGCG-GNPs on the concentration of complement protein C3. The concentration of C3 decreased from 140 ± 2.5 mg/dL to 105 ±1.5 and 110 ± 2.9 mg/dL after incubation with EGCG and EGCG-GNPs, respectively. This decrease in C3 concentration was non-significant when compared with the control (*p* > 0.05). The difference between C3 concentration for EGCG and EGCG-GNPs was also non-significant (*p* > 0.05). The values of C3 were still within normal range (80–160 mg/dL), which confirms the biocompatibility of the prepared EGCG-GNPs [61]. 

### 2.10. In Vivo Studies

**Figure 9:**
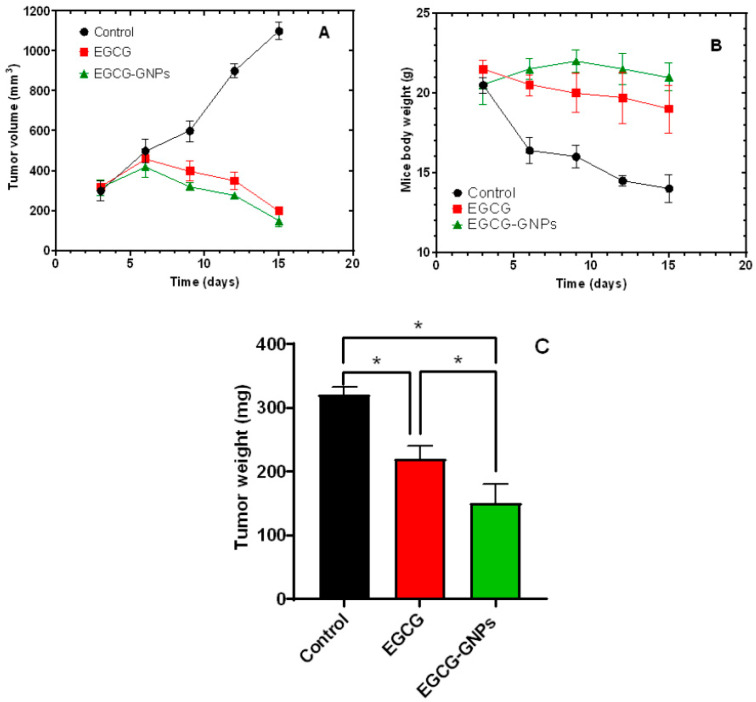
(**A**) Ehrlich ascites carcinoma growth curve. (**B**) Changes in the body weight of the Ehrlich tumor-bearing mice as a function of time. (**C**) Weight of the tumor at the end of the study. * Statistically significant difference (*p* < 0.05). The observed results confirm that loading of EGCG onto GNPs resulted in significant enhancement of its anticancer efficacy.

The in vivo anticancer efficacy of EGCG-GNPs was evaluated in mice bearing Ehrlich ascites carcinoma where the tumor growth was monitored every three days for 15 consecutive days. Figure 9A shows that three days’ post-treatment a tumor volume of around 300 mm^3^ was observed for the three tested groups (i.e., control, EGCG and EGCG-GNPs groups). Subsequently, the tumor volume in the control group continued to grow progressively and by 15 days more than 3.5-fold increase in the volume was recorded. In contrast, the tumor volume of EGCG and EGCG-GNPs groups increased slightly for up to six days’ post-treatment after which there was a gradual and continuous decline in the volume during the 15 days of study. At the end of the study, a tumor volume of 200 ± 19 and 150 ± 30 mm^3^ was detected for EGCG and EGCG-GNPs groups, respectively. The change in tumor volume of the groups treated with EGCG and EGCG-GNPs was highly significant when compared to the control (*p* < 0.05). Further, comparing the tumor volumes of EGCG and EGCG-GNPs groups shows that after 15 days of treatment, the volume in EGCG-GNPs-treated group was significantly smaller than that in EGCG-treated group (*p* < 0.05). During the experiment, there were no abnormal clinical signs on the mice except weight loss of the control group (Figure 9B). At the end of the experiment, the weight of the mice in the control group was very significantly lower than that of either the EGCG or EGCG-GNPs groups (*p* < 0.001). Further, the body weight in EGCG-treated group was significantly smaller than that treated with EGCG-GNPs (*p* < 0.05) (Figure 9B).

After 15 days of treatment, the mice were sacrificed, and the tumors were removed and weighed. Figure 9C shows that the tumor weight in the EGCG-GNPs group was significantly smaller than that of either the control group or EGCG group (*p* < 0.05). The treatment with EGCG and EGCG-GNPs resulted in a 1.45- and 2.13-fold reduction in the tumor weight compared with the untreated control. The enhanced anticancer efficacy observed for EGCG-GNPs compared with EGCG alone might be attributed to several reasons. First, the small size and negative charge of the nanoparticles could help increase their circulation time in the blood and thus enhance their accumulation in tumors through the enhanced permeability and retention effect [43,44]. Second, the observed sustained drug release could help the nanoparticles maintain their cargo until reaching the tumor site, which in turn increases drug accumulation in the tumor. Enhanced anticancer efficacy for EGCG-GNPs was observed previously against several other cancers. Thus, EGCG-GNPs had 4.91-fold higher cytotoxicity against murine B16F10 melanoma cells compared with EGCG, in vitro [7]. In another in vivo study, EGCG-GNPs had significantly lower MBT-2 tumor volume compared with EGCG when given orally or by intraperitoneal injection in a C3H/He mouse model [65]. However, the magnitude of enhancement of the in vivo anticancer effect of EGCG-GNPs compared with EGCG alone was not as pronounced as that observed in the in vitro studies [29]. It is noteworthy that targeting high drug concentrations to the tumor after intravenous administration of drug-loaded GNPs is a very challenging task owing to several barriers that nanoparticles encounter in vivo, such as opsonization and clearance by the reticuloendothelial system, plasma protein adsorption, and aggregation and off-target accumulation. For these reasons some studies opted for direct intratumoral injection of EGCG-GNPs where EGCG-loaded GNPs achieved much better anticancer efficacy compared with the free drug [7,30]. This route of drug administration ensures localized delivery of high drug concentration and subsequent enhanced therapeutic efficacy, however it is applicable only for easily accessible superficial tumors. 

## 3. Materials and Methods

### 3.1. Materials

EGCG was purchased from Nantong Chem-Base Laboratories Co., Ltd., Nantong, China. Gold chloride (HAuCl_4_.H_2_O) was purchased from Electron Microscopy Sciences Co. (Hatfield, PA, USA). Ultra-centrifugal filters (Amicon^®^ Ultra-4 30k Merck Millipore) were obtained from Fisher Scientific, Waltham, MS, USA. Dialysis membranes (MWCO 3.5 kDa) were obtained from Spectrum Laboratories Inc. (Rancho Dominguez, CA, USA). All other chemicals were of reagent grade and used as received. 

### 3.2. Fabrication of EGCG-Loaded Gold Nanoparticles (EGCG-GNPs)

EGCG-GNPs were prepared from chloroauric acid (HAuCl_4_) using EGCG as a reducing and stabilizing agent [25]. Briefly, HAuCl_4_ aqueous solution (2 mL, 0.5 mM) were added to EGCG aqueous solution (8 mL, 0.109–2.618 mM) under magnetic stirring. The color of the solution turned from pale yellow to purple red within 15 min asserting the formation of gold nanoparticles. Different formulations were prepared that had constant HAuCl_4_ concentration and varying concentrations of EGCG according to the composition given in Table 1 so that the EGCG/HAuCl_4_ molar ratio ranged from 0.2:1.0 to 4.7:1.0. The nanoparticles were kept at 4 °C for further analysis. UV-Vis spectra of EGCG-GNPs in the range of 500–700 nm were measured using a UV-Vis spectrophotometer (Shimadzu-50-02, Kyoto, Japan).

### 3.3. Determination of Drug Encapsulation Efficiency

Aliquots of EGCG-GNPs were subjected to ultrafiltration using Amicon^®^ Ultra-4 30k (Merck Millipore, Fisher Scientific, Waltham, MS, USA) for 20 min at 9900 rpm. EGCG concentration in the supernatant was calculated from its spectrophotometric absorbance at λ = 274 nm and using a calibration curve. The percent encapsulation efficiency (EE) and loading capacity (LC) were calculated from Equations (1) and (2), respectively.

(1) $$\mathrm{EGCG}\;\mathrm{EE}\;(\%)\;=\;\frac{\;\mathrm{Initial}\;\mathrm{EGCG}\;\mathrm{concentration}\;-\mathrm{EGCG}\;\mathrm{concentration}\;\mathrm{in}\;\mathrm{the}\;\mathrm{supernatant}}{\;\mathrm{Initial}\;\mathrm{EGCG}\;\mathrm{concentration}}\;\times \;100\;$$

(2) $$\mathrm{EGCG}\;\mathrm{LC}\;(\%)\;=\;\frac{\;\mathrm{Weight}\;\mathrm{of}\;\mathrm{EGCG}\;\mathrm{in}\;\mathrm{GNPs}}{\mathrm{Total}\;\mathrm{weight}\;\mathrm{of}\;\mathrm{tested}\;\mathrm{GNPs}}\;\times \;100\;$$

### 3.4. Particle Size and Zeta Potential Measurements

The particle size, polydispersity index and zeta potential of formulations F1–F6 were measured using a Malvern ZetaSizer (Nano-ZS, Malvern Instruments, Worcestershire, UK). The instrument was equipped with a 4-mW helium/neon laser (λ = 633 nm) and the measurements were done at room temperature. Zeta potential was calculated from the electrophoretic mobility values using the Smoluchowski equation. Each sample was measured three times and the mean ± SD is reported. 

### 3.5. Transmission Electron Microscope (TEM) Measurements

A high-resolution transmission electron microscope (Hitachi-H7650, Santa Clara, CA, USA) was used to characterize the size and morphology of EGCG-loaded GNPs (formulation F2). Aliquot of the sample (100 µL) was added onto a 200-mesh TEM copper grid. After a 5-min drying time at room temperature, excess sample was carefully removed by a piece of filter paper and the sample was stained using aliquot of aqueous uranyl acetate solution (2%, *w*/*v*). The imaging was performed at an accelerating voltage of 80 kV and data acquisition was accomplished using an AMT-700 capture image camera (Advanced Microscopy Techniques, Woburn, MA, USA).

### 3.6. Fourier-Transform Infrared (FT-IR) Spectroscopy Studies

FT-IR studies were carried out using Digilab Spectrum Excalibur FT-IR spectrometer (Digilab, Randolph, MA, USA) equipped with an attenuated total reflectance (ATR) accessory. A dry sample was pressed onto the ATR crystal and the infrared spectrum was recorded from 4000 to 400 cm^−1^ at room temperature and resolution of 4 cm^−1^. The studied samples were EGCG and EGCG-loaded GNPs (formulations F2). 

### 3.7. Differential Scanning Calorimetry (DSC) Studies

The DSC thermograms were obtained using a DSC-50 differential scanning calorimeter (Shimadzu, Seisakusho Ltd., Kyoto, Japan). Samples (3–5 mg) of EGCG, HAuCl_4_ or EGCG-loaded GNPs (formulations F2) were placed in aluminum pans and heated at a scanning rate of 10 °C/min from 30 to 250 °C in the presence of nitrogen at a flow rate of 40 mL/min. Indium standard was used to calibrate the instrument. 

### 3.8. In Vitro Drug Release Studies

EGCG release studies were carried out using the dialysis bag method [36]. Drug-loaded GNPs aqueous solution (3 mL, 0.9 mg EGCG) was introduced into a dialysis bag membrane (MWCO 3.5 kDa). The membranes were put into 25 mL of phosphate buffer pH 7.4 in tightly closed tubes. The tubes were put in a water bath shaker operating at a speed of 50 rpm at a temperature of 37 °C. Aliquots were withdrawn from the release media at different time intervals up to 72 h and replaced by the same volume of fresh medium. Concentration of EGCG in the samples was measured spectrophotometrically at 274 nm and using a calibration curve. Cumulative percent of EGCG released was plotted as a function of time.

### 3.9. Storage Stability Study

Aliquots of the optimized formulation F2 were stored in tightly closed containers at 4 °C and at room temperature for 4 months. At predetermined time intervals (0, 1, 2, 3, 4 months), aliquots were withdrawn and characterized for drug encapsulation efficiency, particle size, polydispersity index, zeta potential, and UV-Vis spectra as described above.

### 3.10. Hemocompatibility Studies

#### 3.10.1. Hemolysis Test

The hemolysis assay was carried out according to the protocol from National Cancer Institute (NCI) [66]. Venous blood samples were collected from male volunteers. This procedure was approved by the institutional ethics committee of Medical Research Institute, Alexandria University, Egypt (approval number E/C. S/N. 10/2020). Blood samples had a plasma free hemoglobin (PFH) concentration below 1.0 mg/mL. Blood samples were collected on Li-heparin as an anti-coagulant [67]. Blood was diluted with phosphate-buffered saline (PBS) pH 7.4 to get total blood hemoglobin (TBH) concentration of 10±2 mg/mL. Each sample was divided into three aliquots each of 100 μL. First aliquot was treated by EGCG-GNPs solution (100 μL, formulation F2 containing 218.16 µM of EGCG). The second aliquot was treated by EGCG aqueous solution (100 μL, EGCG concentration of 218.16 µM). The third aliquot was treated with 100 μL of PBS and served as a control. Similarly treated polyethylene glycol and Triton-X-100 were used as negative and positive controls, respectively. All the mixtures were incubated at 37 °C for 60 min and then centrifuged for 5 min at 3500 rpm. 100 μL of supernatant and 100 μL cyanmethemoglobin (CMH) reagent were added to a 96-well plate. The optical density (OD_test_) of the tested supernatant was measured spectrophotometrically at 540 nm. Percent hemolysis was calculated using Equation (3).

(3) $$\%\;\mathrm{Hemolysis}=\frac{{\mathrm{OD}}_{\mathrm{test}}-{\mathrm{OD}}_{-\mathrm{ve}}}{{\mathrm{OD}}_{+\mathrm{ve}}-{\mathrm{OD}}_{-\mathrm{ve}}}\times 100$$

A test sample having percent hemolysis less than 2% was considered not hemolytic. Background interference was subtracted from OD of each term in the previous equation. 

#### 3.10.2. Partial Thromboplastin Time (PTT) and Prothrombin Time (PT) Assay

Blood samples were collected in sodium citrate tubes then divided into three aliquots each of 2 mL. The first aliquot was incubated with EGCG-GNPs solution (2 mL, formulation F2 containing 218.16 µM of EGCG) for 30 min at room temperature. The second aliquot was incubated with EGCG aqueous solution (2 mL, EGCG concentration of 218.16 µM) for 30 min at room temperature. The third aliquot was not incubated with any solution and used as a control. The anticoagulated blood samples were centrifuged at 2500 rpm for 10 min after incubation and citrated plasma was separated and used in the estimation of PTT and PT. 

For determination of PTT, the tested blood plasma and positive control (glass) were added in six polypropylene tubes. All tubes were then incubated in a water bath for 1 h at 37 °C with agitation. All plasma samples of volume 50 μL were then transferred to new test tubes and incubated in ice until the PTT values were estimated by recording the time of clot formation. The negative control (untreated plasma) was prepared with the same procedure. The same procedure was applied to the three aliquots in order to determine the PTT for all samples. For PT determination, 0.2 mL of thromboplastin-calcium mixture was added into a set of six test tubes and warmed to 37 °C for 1 min on heat block. Citrated plasma was incubated in water bath until it reached 37 °C for no more than 10 min. Citrated plasma was then added to the thromboplastin-calcium mixture and the time of clot formation was determined using a stopwatch [68]. 

#### 3.10.3. Complement Activation by the Nephelometric Method

Complement activation by EGCG and EGCG-GNPs was done by the nephelometric method by estimating the reduction of complement factor C3. Three aliquots of citrated plasma each of 100 µL were incubated with EGCG, EGCG-GNPs, and saline respectively. The incubation was done for 1 h at 37 °C under gentle agitation. Citrated plasma incubated with normal saline and polyethyleneimine solution (PEI) was used as a negative and positive controls, respectively. C3 concentration was determined by comparison with the known concentration calibrator. The analysis was performed according to the kit’s manufacturer manual. An aliquot of plasma (10 µL) was mixed with antihuman C3 antibody and absorbance was read at 340 nm using a UV–Vis spectrophotometer [69].

### 3.11. In Vivo Studies

This study was conducted on male mice weighing 20–25 g and of age 4–5 weeks. The study was approved by the Ethical Review Board of Faculty of Pharmacy, Assiut University, Egypt (approval number S9-20, 6 February 2020) and it adhered to the National Institutes of Health guide for the care and use of laboratory animals (NIH Publications No. 8023, revised 1978). All animals were housed in stainless steel cages at room temperature (20–26 °C) and humidity of 30–70%. Induction of Ehrlich’s tumor was developed by injection of 2 million tumor ascites cells in 100 μL PBS subcutaneously in the thigh of each mouse. Tumor growth was monitored post-inoculation until the volume was about 200–500 mm^3^. A total of 90 mice were used and were divided randomly into three groups, each of 30 mice. Group A was intravenously injected with 0.2 mL of PBS each 3 days for 15 days and was used as a control. Group B was injected with EGCG aqueous solution (0.2 mL, EGCG concentration of 218.16 µM). Group C was injected with EGCG-GNPs aqueous solution (0.2 mL, formulation F2 containing 218.16 µM of EGCG). EGCG dose in group B and C was 1 mg/kg of mouse body weight each 3 days for 15 consecutive days by intravenous injection. 

The ellipsoidal tumor volume (V) was calculated from Equation (4).

(4) $$V=\frac{\pi }{6}\times D\;\times d^{2}$$

where D is the tumor dimension at the longest point and d is the tumor dimension at the widest point. D and d were measured with a digital caliper.

The tumor volume and mouse total body weight were monitored every three days for all groups. The first day of injection was considered as day 1. By the end of the experiment, all mice were euthanized, and the tumors were completely removed and weighed. 

### 3.12. Statistical Analysis

Results of all the experiments are expressed as mean ± SD of at least three independent measurements. Data were statistically analyzed using GraphPad Prism software version 8.0.1 (GraphPad Software Inc., La Jolla, CA, USA). One-way analysis of variance (ANOVA) with Newman–Keuls post-hoc test was used to test the differences. Statistical significance was defined as *p* < 0.05.

## 4. Conclusions

EGCG-loaded GNPs were prepared using a simple and efficient green procedure where EGCG served a dual role; a reducing and stabilizing agent and the drug itself. Successful formation of EGCG-GNPs was ascertained by the presence of characteristic surface plasmon resonance peaks at λmax of 531 nm and a size in the range of ~26–610 nm. The drug delivery attributes of EGCG-GNPs, such as particle size, drug encapsulation efficiency, and loading capacity were greatly influenced by the drug/gold chloride molar ratio. This ratio also affected the formation of the nanoparticles where ratios larger than 1.8:1 resulted in nanoparticle aggregation. The optimum formulation showed high drug encapsulation efficiency and loading capacity of 92.6 ± 0.6% and 31.6 ± 0.1%, respectively. FT-IR and DSC studies confirmed the loading of EGCG onto the surface of GNPs. The nanoparticles were able to sustain EGCG release for more than 3 days. Upon incubation with the human blood EGCG-GNPs were free of deleterious effects on the blood hemolysis, coagulation system, and protein component C3, which confirms their hemocompatibility. Storage stability studies showed that EGCG-GNPs had acceptable changes in the drug encapsulation efficiency, size, and zeta potential after storage at 4 °C, and room temperature for three months. Further, the nanoparticles had significantly higher cytotoxic effects in vivo in Ehrlich tumor-bearing mice when compared with free EGCG. Taken together, these results confirm that EGCG-GNPs could establish themselves as an eco-friendly nanocarrier system with a good potential to enhance EGCG anticancer properties. Future directions of this work should focus on the in vivo biodistribution of EGCG-GNPs, how they are accumulated in the tumor, and how the body get rid of them. 

## Figures and Tables

**Table 1:** Composition and properties of various EGCG-loaded GNPs.

Formula	EGCG/HAuCl_4_^a^	EE (%) ^b^	LC (%) ^c^	Z- Average Size (nm) ^d^	PDI ^e^
F1	0.2:1	72 ± 7.7	15.2 ± 1.4	26.3 ± 2.4	0.55 ± 0.01
F2	0.4:1	92.6 ± 0.6	31.6 ± 0.1	33.8 ± 2.2	0.33 ± 0.04
F3	0.8:1	32.5 ± 5.4	24.4 ± 3.1	43.8 ± 1.7	0.46 ± 0.02
F4	1.6:1	18.1 ± 0.4	26.6 ± 0.4	332.9 ± 10.5	0.57 ± 0.03
F5	3.1:1	26.1 ± 2.2	51 ± 2.1	56.9 ± 3.6	0.42 ± 0.05
F6	4.7:1	26.9 ± 13.9	58.8 ± 13.9	610.1 ± 20.7	0.78 ± 0.10

^a^ EGCG/HAuCl_4_ molar ratio. ^b^ Percent EGCG encapsulation efficiency determined using equation 1. ^c^ Percent EGCG loading capacity determined using equation 2. ^d^ Z-average particle size. ^e^ Polydispersity index. All the data are presented as the mean of three different measurements ± SD.

**Table 2:** Effect of storage time and temperature on the drug encapsulation efficiency (%), particle size, polydispersity index and zeta potential of EGCG-GNPs formulation F2.

Time	Encapsulation Efficiency (%)		Z- Average Size (nm)		PDI		Zeta Potential (mV)	
	4 °C	RT	4 °C	RT	4 °C	RT	4 °C	RT
**Zero time**	92.6 ± 0.6	92.6 ± 0.6	33.8 ± 2.2	33.8 ± 2.2	0.3 ± 0.04	0.3 ± 0.04	−24.9 ± 0.3	−24.9 ± 0.3
**1 month**	90.8 ± 0.4	89.7 ± 0.2 *	37.9 ± 1.4 *	32.3 ± 0.9	0.4 ± 0.0	0.4 ± 0.1	−27.1 ± 1.3 *	−23.3 ± 1.4 *
**2 months**	90.5 ± 0.6	86.7 ± 0.2 *	30.3 ± 0.8 *	30.3 ± 0.3	0.3 ± 0.0	0.3 ± 0.0	−25.4 ± 0.8	−27.1 ± 1.0 *
**3 months**	91.3 ± 1.6	89.7 ± 0.8 *	44.2 ± 0.3 *	30.7 ± 1.9	0.4 ± 0.0	0.3 ± 0.1	−28.0 ± 0.6 *	−28.3 ± 0.3 *
**4 months**	86.7 ± 1.2 *	81.1 ± 1.6 *	33.7 ± 1.0	31.8 ± 0.5	0.3 ± 0.0	0.3 ± 0.0	−25.1 ± 0.4	−26.1 ± 0.7

RT: room temperature, PDI: polydispersity index. All values are reported as mean ± SD of three different measurements. *: Statistically significant difference compared to the value at zero time (*p* < 0.05).
